# Evaluating impulse control disorders in people with Parkinson’s disease: agreement and added value of patient and caregiver reports

**DOI:** 10.3389/fneur.2026.1791205

**Published:** 2026-04-30

**Authors:** Sara C. Staubo, Elisabet Størset, Mathias Toft, Ingeborg H. Lie, Kirsti M. J. Alvik, Pål Jostad, Stein H. Tingvoll, Kristina Rosqvist, Per Odin, Erik Sveberg Dietrichs, Espen Dietrichs

**Affiliations:** 1 Department of Neurology, Oslo University Hospital, Oslo, Norway; 2 Institute of Clinical Medicine, University of Oslo, Oslo, Norway; 3 Department of Neurology, Akershus University Hospital, Nordbyhagen, Norway; 4 Center for Psychopharmacology, Diakonhjemmet Hospital, Oslo, Norway; 5 Unicare Fram Rehabilitation Centre, Rykkinn, Norway; 6 Ringen Rehabilitation Centre, Moelv, Norway; 7 Division of Neurology, Department of Clinical Sciences, Lund University, Lund, Sweden; 8 Department of Neurology, Rehabilitation Medicine, Memory and Geriatrics, Skåne University Hospital, Lund, Sweden; 9 Institute for Experimental Medical Research, University of Oslo and Oslo University Hospital, Oslo, Norway; 10 Experimental and Clinical Pharmacology, Institute of Medical Biology, UiT The Arctic University of Norway, Tromsø, Norway

**Keywords:** caregivers, dopamine agonist, impulse control disorders, Parkinson’s disease, QUIP-RS

## Abstract

**Background:**

People with Parkinson’s Disease (PD) treated with dopamine agonists are at increased risk for developing impaired impulse control behavior. It has been suggested that patients tend to underreport problems with impaired impulse control, indicating that caregiver evaluations may be a helpful supplement to clinical assessment.

**Objective:**

To examine the agreement between evaluations of impaired impulse control provided by people with PD treated with dopamine agonists and by their caregivers.

**Methods:**

People with PD and their caregivers independently completed the validated Questionnaire for Impulse-Compulsive Disorders in Parkinson’s Disease-Rating Scale (QUIP-RS) and the Impulse Control Disorders and Related Conditions (ICDRC) questionnaire. Patient and caregiver assessment were compared statistically for total scores and subscores on both questionnaires.

**Results:**

Overall, patient and caregiver assessment on both the QUIP-RS and ICDRC showed no consistent systematic differences. Patients reported a slightly higher total ICDRC score and higher combined subscores for punding and dopamine dysregulation syndrome compared to their caregivers. Further, patient evaluations showed a wider range of responses for hypersexuality (QUIP-RS and ICDRC) and for gambling (ICDRC).

**Conclusion:**

The assessment of impaired impulse control remains difficult, and our findings indicate that the role of caregiver evaluations is still uncertain.

## 1 Introduction

Dopamine agonist treatment is a significant risk factor for the development of impaired impulse control in people with Parkinson’s disease (PD). A large cohort study reported that 17% of people with PD using dopamine agonists have impaired impulse control (1), while a longitudinal study revealed that over 50% will experience periods of impaired impulse control within 5 years (2). The association between dopamine agonists and impulse control disorders was first published in 2006 (3), several years after non-ergot dopamine agonists were introduced to treat motor symptoms in people with PD (4). It remains unclear why the negative side effects of impaired impulse control were not recognized until then. One study that compared spontaneous patient reports and structured interviews found that few patients voluntarily reported impaired impulse control (5). It has also been suggested that people with PD tend to underreport these problems (6–8), indicating a higher prevalence of impulse control problems than initially thought.

Impaired impulse control is characterized as a behavioral disorder where patients fail to resist an impulse with compulsive features, negatively impacting themselves or their environment (9–12). Classic impulse control disorders (ICD) include pathological gambling, hypersexuality, compulsive shopping and binge eating (9). Additionally, hobbyism, punding and dopamine dysregulation syndrome are often observed in people with PD and therefore included in the term “Impulse-control disorders and related behavioral disorders” (10, 12).

To address the underreporting of impaired impulse control a specialized questionnaire has been developed (7). This is the foundation for the validated Questionnaire for Impulse-Compulsive Disorders in Parkinson’s Disorder in Parkinson’s Disease-Rating Scale (QUIP-RS), which also assesses severity (8) and is the only validated questionnaire for impaired impulse control to date. It has been suggested that a caregiver also should be included in the assessment of impaired impulse control (13), since this can contribute to detection.

One previous study compared the assessments based on information given by people with PD and information given by their caregivers, regarding impaired impulse control (14). Using the Impulse Control Disorders and Related Conditions (ICDRC) questionnaire, Baumann-Vogel et al. found that people with PD underreport the presence of problems with hypersexuality, punding and dopamine dysregulation syndrome, compared to their caregivers (14). In the present study, we aim to compare evaluations provided by people with PD that are treated with either pramipexole or ropinirole, and evaluations given by their caregivers. This is achieved by utilizing the validated QUIP-RS screening tool to assess impulse control issues, while also incorporating results from the ICDRC questionnaire for comparison with the findings of Baumann-Vogel et al.

## 2 Materials and methods

### 2.1 Design

This study is a preplanned secondary analysis based on the dataset of the ICD Parkinson Agonist Pharmacology Study (IPAPS) (15, 16)—a clinical and pharmacological Scandinavian multicenter study with a cross-sectional observational design. In the planning phase of IPAPS, we recognized that assessing impaired impulse control disorders in Parkinson’s disease is challenging and susceptible to underreporting, and we therefore decided to include the present secondary analysis. While the primary aim of IPAPS was to explore the potential correlation between dopamine agonist serum concentrations and impaired impulse control in people with PD, the evaluation of agreement between patient and caregiver reports was predefined as a secondary objective. Participants underwent detailed clinical interviews, completed standardized forms, underwent clinical examination, and provided three blood samples within a 12-hour period. More detailed methodology is available in our previous articles (15, 16).

The study was approved by the Regional Ethical Committee in Northern Norway (reference: 2018/1343/REK nord), the Personvernombudet/Datatilsynet (General Data Protection Regulation in Norway; reference: 2018/6255), and the Swedish Ethical Board (reference: 2022–01340–01).

### 2.2 Participants

Recruitment took place from spring 2020 to fall 2022, involving four centers in Norway (Oslo University Hospital, University Hospital of Northern Norway, Ringen Rehabilitation Center and Unicare Fram Rehabilitation Center) and one center in Sweden (Skåne University Hospital, Lund). The inclusion criteria were a diagnosis of idiopathic PD as defined by the International Parkinson and Movement Disorder Society clinical diagnostic criteria (17), ongoing treatment with either pramipexole or ropinirole extended release in the morning, and no change in dopaminergic treatment during the last month. Exclusion criteria included cognitive impairment. We did not perform formal cognitive tests, but all patients were assessed in clinical interviews and only those without any signs of cognitive impairment were included. Participants were included irrespective of their impulse control status. Informed consent was obtained from each study participant before inclusion in the study.

Participants are referred to as patients, participants and people with PD throughout the text.

### 2.3 Assessment of impaired impulse control

To evaluate the level of impulse control, we used the QUIP-RS (8) and ICDRC (14). Both patients and caregivers were invited to complete the questionnaires. Caregivers were identified by the patients themselves (typically a spouse, partner or close family member) and were asked to complete the questionnaire independently. Patients filled out the standard version of each questionnaire, and caregivers were given slightly adapted forms, replacing “you” by “he” or she.” Although only the pronouns were changed and the item content and response options remained the same, these adapted caregiver versions have not been formally validated, and the caregiver results should therefore be interpreted with some caution. Both questionnaires include five response options for each question. The QUIP-RS has the following alternatives: “never,” “rarely,” “sometimes,” “often,” and “very often.” The ICDRC has the following options: “never,” “rarely,” “sometimes,” “very often,” and “always.” Responses from the questionnaires were transformed from the original five-point scales into ordinal scales by assigning numeric values ranging from 0 to 4.

We analyzed the total QUIP-RS score and the separate subscores A-G for gambling (A), hypersexuality (B), compulsive shopping (C), binge eating (D), hobbyism (E), punding (F) and dopamine dysregulation syndrome (G), respectively (8). Validated cut-off scores defining each ICD (8) were used to compare reports from patients and caregivers. For the ICDRC, the total score and individual subscores for gambling, hypersexuality, compulsive shopping, binge eating, punding and dopamine dysregulation syndrome were evaluated.

### 2.4 Further assessments

Participants underwent a detailed neurological examination, which included a complete assessment of motor functions using the MDS-Unified Parkinson’s Disease Rating Scale (MDS-UPDRS) parts III (ON medication) and IV (18), as well as the Hoehn and Yahr rating scale (19, 20). In addition to the motor assessment, participants were thoroughly interviewed and completed several questionnaires, including the Non-Motor Symptoms Questionnaire (NMSQ) (21) and the Parkinson’s Disease Questionnaire (PDQ-39) for health-related quality of life assessment (22). Further details have been provided in our previous articles (15, 16).

### 2.5 Statistical analysis

Our data were transformed from a 5-point scale to an ordinal scale of 0 to 4. All reported values are reported as medians with interquartile ranges. Mean values are also given for descriptive purposes.

The evaluation scores from patients and their caregivers were visually inspected using scatterplots. Wilcoxon signed-rank test was used to compare the evaluation scores from patients to that of their caregivers. To compare patients who reported lower score on total QUIP-RS than their caregiver with those who reported higher score than their caregiver, we used chi-square tests for gender and dopamine agonist usage and Wilcoxon rank sum tests for the remaining variables. Agreement between the reported scores were assessed using Bland–Altman analysis. *The limits of agreement were calculated by using the formula of ±1.96 times the standard deviation of the mean differences. p*-values below 0.05 indicated statistically significant differences.

Statistical analyses were performed in STATA/SE 18.0 and graphics were developed in STATA/SE 18.0 and MedCalc (version 23.4).

## 3 Results

### 3.1 Participant characteristics

Complete data from both patient and caregiver were available from 42 pairs. The characteristics of these are presented in Table 1. We have also compared them to the remaining 58 IPAPS participants who lacked complete caregiver data. Analyses revealed no significant differences between the two groups regarding subscores and total scores on the QUIP-RS and ICDRC assessments. The underlying reason why fewer than half of the participants provided caregiver information are not fully known, although the fact that caregiver participation was voluntary likely contributed. A number of patients lived alone and reported the absence of close caregivers to invite, but their number was not recorded. Also, by mistake caregiver evaluation forms were not distributed from one of the five IPAPS study sites.

**Table 1:** Patient characteristics.

Characteristics	All patients with data from caregiver	
Number of participants	42	
Gender	27 male; 15 female	
Ropinirole users	22 (15 male; 7 female)	
Pramipexole users	20 (12 male; 8 female)	
	*Median*	*Interquartile range*
Age (years)	63	56–69
Time since PD symptom debut (months)	81	60–108
Time with dopaminergic treatment (months)	49	24–80
Time with dopamine agonist treatment (months)	53	33–81
Total LEDD (mg)	757	473–862
LEDD dopamine agonist (mg)	172	147–291
Hoehn and Yahr stage	2.0	1.5–2.5
MDS-UPDRS III	17	9–26
MDS-UPDRS IV	1	0–4
MDS-UPDRS IV 4.1–4.2	0	0–1
MDS-UPDRS IV 4.3–4.6	0.5	0–3
PDQ39- SI	33	25–39
NMSQ	10	6–13

LEDD, Levodopa Equivalent Daily Dose; MDS-UPDRS, Movement Disorders Society Modified Unified Parkinson’s Disease Rating Scale; PDQ39-SI, Parkinson’s Disease Questionnaire Summary Index; NMSQ, Non-Motor Symptoms Questionnaire.

Dopaminergic treatment burden was characterized by total LEDD and LEDD for dopamine agonists. Motor symptoms severity and motor complications were assessed using the MDS-UPDRS III and IV. Scores on MDS-UPDRS IV items 4.1–4.2 and 4.3–4.6 were generally low, indicating a relatively modest burden of dyskinesias and motor fluctuations in this sample.

### 3.2 Comparison of patients and caregiver assessments

**Figure 1:**
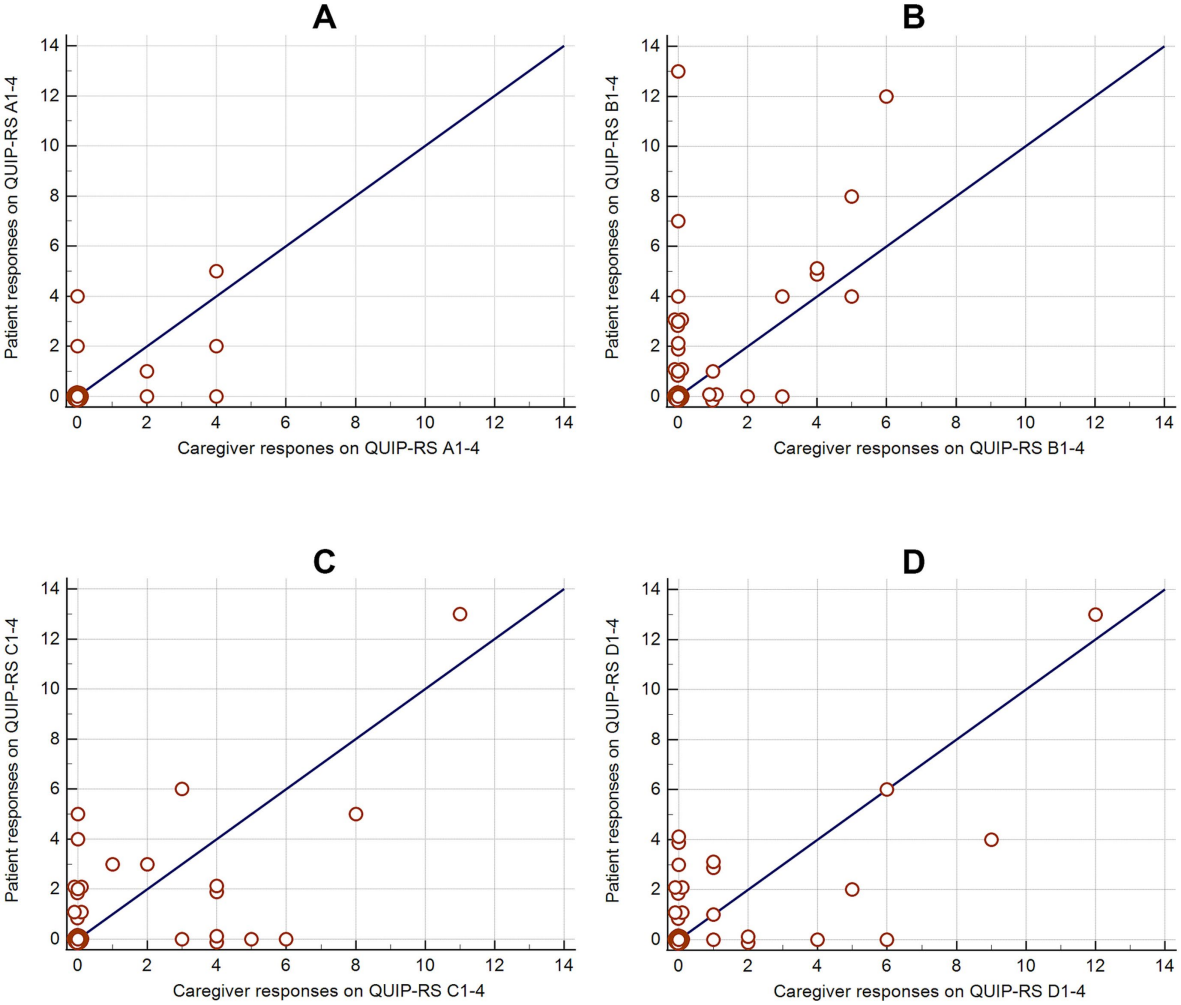
Comparison of patient and caregiver responses on QUIP-RS subcategories **(A–D)** illustrated with scatter plots and lines of unity. Subcategory **A** refers to gambling, **B** refers to hypersexuality, **C** refers to compulsive shopping, and **D** refers to binge eating.

In the QUIP-RS questionnaire, the patient and caregiver assessments were not significantly different, except from a difference in the hypersexuality subscore (*p* = 0.007, B1-4; Table 2; Figure 1). Although both groups had the same median score for hypersexuality, the distribution of scores differed. Caregiver assessments were more concentrated, with values reaching a maximum of 6, indicating a tighter clustering around the median. In contrast, patient assessments exhibited a wider spread of values, with scores reaching up to 13. Apart from this difference, the two groups were comparable in their QUIP-RS assessments.

**Table 2:** Comparison of patient and caregiver data.

Domain (items)	Patient			Caregiver			*p*
	Median	Mean	Interquartile range	Median	Mean	Interquartile range	
QUIP-RS							
Gambling (A1-4)	0	0.33	0–0	0	0.38	0–0	0.83
Hypersexuality (B1-4)	0	1.98	0–3	0	0.86	0–1	0.007
Compulsive shopping (C 1–4)	0	1.29	0–2	0	1.31	0–2	0.52
Binge eating (D 1–4)	0	1.24	0–2	0	1.19	0–1	0.38
Hobbyism, punding, dopamine dysregulation syndrome (E-G)	2	3.88	0–5	2	4.69	0–8	0.60
Total QUIP-RS	5	8.71	1–12	4	8.43	0.13	0.65
ICDRC							
Gambling (1-2)	0	0.48	0–0	0	0.19	0–0	0.02
Hypersexuality (1-2)	0	0.90	0–1	0	0.48	0–1	0.02
Compulsive shopping (1-2)	0.5	0.93	0–2	0	0.64	0–1	0.11
Binge eating (1-2)	0	0.93	0–1	0	1.1	0–2	0.82
Punding, dopamine dysregulation syndrome (E-F)	3	3.21	1–4	2	2.38	0–4	0.005
Total ICDRC	5	6.45	3–9	3.5	4.76	0–7	0.0006

Wilcoxon signed-rank test was used to compare the data from patients and caregivers. QUIP-RS, Questionnaire for Impulsive-Compulsive Disorders in Parkinson’s Disease-Rating Scale; ICDRC, Impulse Control Disorders and Related Conditions.

In the ICDRC questionnaire, there was a significant difference in patients and caregivers assessments for gambling and hypersexuality, despite identical medians (*p* = 0.02; Table 2). The patient groups demonstrated a broader distribution in comparison to caregivers. For the summed subscores (E-F) and the total ICDRC score, caregiver assessments revealed a significantly lower median value compared to the patient assessments (*p* = 0.005, *p* = 0.0006). Some outliers were present in the caregiver data, but they did not significantly alter this central tendency.

Five of our patients scored above the QUIP-RS validated cut-offs for classic ICD, three for hypersexuality, one for compulsive shopping and one for binge eating (Table 3). The caregivers also scored above cut-off for the two latter, but not for the three first. One caregiver scored above cut-off (both for compulsive shopping and binge eating) in a patient that scored below. None of the patients or caregivers scored above the cut-off for gambling.

**Table 3:** The table shows the six pairs of participant/caregiver that scored above the ICD cutoff for one or more of the QUIP-RS subcategories.

Participant-caregiver pair	Gambling	Hypersexuality	Compulsive shopping	Binge eating
	Participant/caregiver	Participant/caregiver	Participant/Caregiver	Participant/caregiver
1		+/0		
2		+/0		
3		+/0		
4			+/+	
5			0/+	0/+
6				+/+

Participant-caregiver pairs are listed in the first column. Scores above the cutoff are indicated with a ‘+’, while scores below the cutoff are marked with a ‘0’.

**Figure 2:**
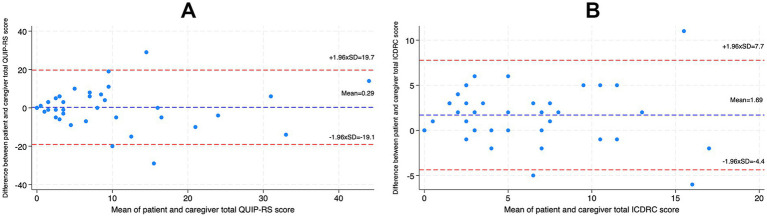
Bland–Altman plot comparing responses from patients and their caregivers on QUIP-RS **(A)** and ICDRC **(B)**. The *y*-axis represents the difference between patient responses and caregiver responses (Patient - caregiver), while the *x*-axis shows the mean of the two responses. The solid line indicates the mean difference, and the dashed lines represent the ±1.96 standard deviations from the mean difference, indicating the limits of agreement.

The Bland–Altman analysis of the QUIP-RS revealed a mean difference of 0.29, with 95% limits of agreement ranging from −19.1 to 19.7 (Figure 2A). For the ICDRC, the mean difference was 1.69, with limits of agreement between −4.4 and 7.7 (Figure 2B). The analysis for QUIP-RS indicated that a greater number of patients tended to score lower than their caregivers as mean scores increased. There was no apparent systematic bias for the ICDRC.

### 3.3 Comparison of participants reporting scores below and above their caregivers on QUIP-RS

Our data showed that 20 participants reported total scores higher than their caregivers, while 17 participants scored lower than their caregivers. Five participants reported identical total QUIP-RS scores to their caregivers.

Patients who reported a higher total QUIP-RS score compared to their caregivers had significantly higher scores on the NMSQ (Table 4). Otherwise, the two groups were comparable.

**Table 4:** Comparison of participants reporting scores below and above their caregivers on QUIP-RS.

Characteristic	Patients who reported lower score on total QUIP-RS than caregiver (number or median)	Patients who reported higher score on total QUIP-RS than caregiver (number or median)	*p*-value
Number of participants	17	20	
Gender of participants	11 male; 6 female	14 male; 6 female	0.73
Dopamine agonist	9 ropinirole; 8 pramipexole	10 ropinirole; 10 pramipexole	0.86
Time with dopaminergic treatment (months)	48	62	0.13
Time with dopamine agonist treatment (months)	48	69	0.16
Total LEDD	754	795	0.64
LEDD dopamine agonist	240	192	0.86
Hoehn and Yahr stage	2	2	0.96
MDS-UPDRS III	12	17	0.31
MDS-UPDRS IV	0	3	0.12
PDQ39-SI	29	37	0.17
NMSQ	7	12	0.01

For gender and dopamine agonist usage, we conducted chi-square tests for comparison, while Wilcoxon rank sum tests were employed for analyzing the remaining values. LEDD, Levodopa Equivalent Daily Dose; MDS-UPDRS, Movement Disorders Society Modified Unified Parkinson’s Disease Rating Scale; PDQ-39, Parkinson’s Disease Questionnaire; NMSQ, Non-Motor Symptoms Questionnaire.

## 4 Discussion

Our primary finding is that among our participants we found no systematic difference between patient and caregiver assessments of impaired impulse control. Individual responses did differ, but discrepancies were balanced in both directions and the overall results were similar. Patients who scored above or below their caregivers were largely comparable, with the exception that patients who scored higher than their caregivers had a greater burden of non-motor symptoms according to the NMSQ.

Only minor differences appeared, with patients reporting a somewhat higher occurrence of impaired impulse control compared to their caregivers. Specifically, on the total ICDRC score, patient responses resulted in a significantly higher score than those of caregivers. This was also true for the combined subscores for punding and dopamine dysregulation syndrome on the ICDRC. Patients exhibited a markedly wider range of responses regarding hypersexuality on both the QUIP-RS and the ICDRC. This discrepancy was also evident in the responses related to gambling on the ICDRC.

Somewhat similar observations were made when the validated QUIP-RS cut-off scores for defined ICD were applied. Five patients scored above cut-off for one ICD, but the matching caregiver scored above cut-off for only two of these. Only one caregiver scored above cut-off for a patient who did not, but in this case for two different ICD.

These findings are in contrast with a previous study applying the ICDRC for comparing 64 patients and caregivers (14). They reported a higher prevalence of hypersexuality, punding, and dopamine dysregulation syndrome when assessed by caregivers rather than patients. Compared to the study population of Baumann-Vogel et al., our participants had a shorter mean disease duration (7.6 years compared to 11.8 years) (14). This may in part be an explanation for different results, as longer disease duration is an additional risk factor for developing impaired impulse control in people with PD (12, 23). Further, Baumann-Vogel et al. reported higher mean scores for the total ICDRC score in their patients, with a mean value of 8.6 compared to our 6.5 (14). Previous research has suggested that cultural and socioeconomic factors can influence the perception and reporting of impaired impulse control in people with PD (6, 23, 24). Such dissimilarities may certainly be of importance, but we consider it less likely that they fully explain the different results in between our Scandinavian participants and the Swiss PD patients examined by Baumann-Vogel et al. (14). Another possibility is that increased awareness of impaired impulse control may have led to different study findings in recent studies compared to older ones.

We compared QUIP-RS and ICDRC scores for each participant and their caregiver. Participants more frequently scored higher than their caregivers. The mean difference between the measures of patients and their caregivers was close to zero, suggesting no systematic bias between the two measures. However, we observed a small discrepancy at the higher values of the mean total QUIP-RS, where a greater number of patients scored lower than their caregivers. This finding contrasts with the rest of our results and may be due to random variation. Due to our small dataset, further statistical testing of this finding was not possible. Importantly, the wide limits of agreement in the Bland–Altmann analyses show that agreement at the individual level was variable, with some patient-caregiver pairs differing considerably in their assessments. This indicates that, although there is no systematic difference at the group level, patient and caregiver reports are not interchangeable for individual patients. Furthermore, agreement between patients and caregivers does not necessarily imply accurate detection of all impulse control problems, as both groups may underreport these problems.

Despite this latter observation it is noteworthy that the very highest patient scores, including most of those above cut-offs for an ICD, were not matched by their caregivers. This could, speculatively, indicate a tendency that people with the most severe impulse control issues try to conceal their problems from caregivers and possibly also doctors, potentially driven by feelings of shame or a reluctance to reduce or discontinue dopamine agonist treatment. Patients generally prefer periods in which dopaminergic treatment is effective and motor symptoms are reduced (on) to periods in which medication effects have worn off and symptoms reappear (off), and therefore seek higher doses of dopaminergic medication to avoid the adverse symptoms associated with an off-state. Patients with dopamine dysregulation syndrome may apply pressure on their physicians for “on-demand” medications and request dosage increases even when their motor control is satisfactorily managed (12). This may be relevant to our population, as reflected by the slightly higher mean levodopa equivalent daily dose (LEDD) of dopamine agonists of 208 mg among our participants, compared to a mean LEDD of dopamine agonists of 188 mg reported for the study population of Baumann-Vogel et al. (14).

Our study has several weaknesses. The most obvious is the low number of participants and the resulting weak statistical power. Unfortunately, we were able to collect caregiver data from less than half of our 100 IPAPS participants. We did not systematically assess reasons why caregivers did not participate, and this may have introduced selection bias. Another weakness is the suspected problem with underreporting from the patients. Since our patients and their caregivers scored similarly, it appears that underreporting was not common among our patients, or that underreporting occurred among both patients and caregivers. Alternatively, some patients may possibly both underreport on the study forms and manage to hide their impulse control problems from their caregivers. Some coauthors of this study have observed more severe impulse control problems than those reported by the patients and caregivers in the personal interviews and study questionnaires, but this has not been systematically explored. We did not record patients´ motor state (on vs. off) at the time of questionnaire completion, which is a limitation because impaired impulse control is dopaminergic state-dependent. However, the generally low MDS-UPDRS part IV scores on dyskinesias (items 4.1–4.2) and motor fluctuation/off phenomena (items 4.3–4.6) suggest that motor complications were generally mild in this cohort. Additionally, most patients were in Hoehn and Yahr stage 2, and none above stage 4, so the results may not extend to patients in later disease stages.

A major strength of this study is the use of QUIP-RS, which is the only validated questionnaire for assessing impaired impulse control to date (8). Possible underreporting may impair diagnostic precision when using self-completed questionnaires, but underreporting may also influence an interview-based diagnosis. All PD participants in the IPAPS underwent personal clinical interviews, but these did not warrant re-diagnosis in any of our patients. We diagnosed one or more ICD in 20% of the IPAPS participants, and this figure is comparable with other studies (16). The caregivers in the present study were not interviewed individually, but despite the reservations made above, we still consider the QUIP-RS to be the best diagnostic tool available.

In conclusion, our study revealed no systematic differences between the patients’ and caregivers’ assessments of impaired impulse control, but agreement at the individual level was variable and patient and caregiver reports were not interchangeable for individual patients. However, the evaluation of impulse control problems remains a complex and challenging task. This underscores the need for thorough assessments over time, in bigger study populations than have been evaluated so far, and hopefully with improved diagnostic procedures.

## Data Availability

The raw data supporting the conclusions of this article will be made available by the authors, without undue reservation.
