# Supplementary material for: Evaluating impulse control disorders in people with Parkinson’s disease: agreement and added value of patient and caregiver reports
Source: Front Neurol. 2026 Apr 30;17:1791205. doi: 10.3389/fneur.2026.1791205 (PMC13171319; doi:10.3389/fneur.2026.1791205)
Supplement: Supplementary file 1 [file Table_1.DOCX]

*Table comparing participants with data from caregiver and those without. Wilcoxon signed-rank test was uses to compare the data from participants with data from caregiver and those without.*

|  | **Participants with data from caregiver** | | **Participants without data from caregiver** | | **p-value** |
| --- | --- | --- | --- | --- | --- |
| Number of participants | 42 | | 58 | |  |
| Gender | 27 male; 15 female | | 29 male; 29 female | | 0.16 |
| Dopamine agonist | 22 ropinirole; 20 pramipexole | | 44 ropinirole; 14 pramipexole | | 0.01 |
|  | Median | Interquartile range | Median | Intequartile range |  |
| Age | 63 | 59-69 | 63 | 55-70 | 0.96 |
| Time since symptom debut (months) | 81 | 60-108 | 108 | 61-156 | 0.09 |
| Time with dopaminergic treatment (months) | 48.5 | 24.0-80.3 | 73 | 26-120 | 0.05 |
| Time with dopamine agonit treatment (months) | 52.5 | 33-81 | 72 | 36-100 | 0.24 |
| Total LEDD | 757 | 473-862 | 721 | 530-924 | 0.90 |
| LEDD dopamine agonist | 541 | 263-700 | 500 | 400-730 | 0.88 |
| Hoehn and Yahr stage | 2.0 | 1.5-2.5 | 2.0 | 1.5-2.5 | 0.38 |
| MDS-UPDRS III | 17 | 9-26 | 16 | 12-25 | 0.70 |
| MDS-UPDRS IV | 1 | 0-4 | 1 | 0-4 | 0.27 |
| PDQ39-SI | 33 | 25-39 | 33 | 28-44 | 0.49 |
| NMSQ | 10 | 6-13 | 8 | 6-12 | 0.29 |
| QUIP-RS A1-4 | 0 | 0-0 | 0 | 0-0 | 0.09 |
| QUIP-RS B1-4 | 0 | 0-3 | 1.5 | 0-3 | 0.12 |
| QUIP-RS C1-4 | 0 | 0-2 | 0 | 0-3 | 0.13 |
| QUIP-RS D1-4 | 0 | 0-2 | 0 | 0-3 | 0.17 |
| QUIP-RS E-G | 2 | 0-5 | 2 | 0-8 | 0.23 |
| Total QUIP-RS | 5 | 1-12 | 8 | 2-19 | 0.17 |
| ICDRC A1-2 | 0 | 0-0 | 0 | 0-0 | 0.74 |
| ICDRC B1-2 | 0 | 0-1 | 1 | 0-2 | 0.30 |
| ICDRC C1-2 | 1 | 0-2 | 0 | 0-1 | 0.16 |
| ICDRC D1-2 | 0 | 0-1 | 1 | 0-2 | 0.62 |
| ICDRC E-F | 3 | 1-4 | 3 | 2-4 | 0.97 |
| Total ICDRC | 5 | 3-9 | 7 | 2-11 | 0.77 |
